# Editorial: Molecular Mechanisms Protecting against Tissue Injury

**DOI:** 10.3389/fphar.2016.00272

**Published:** 2016-08-29

**Authors:** Frank A. D. T. G. Wagener, Stephan Immenschuh

**Affiliations:** ^1^Department of Orthodontics and Craniofacial Biology, Radboud University Medical CenterNijmegen, Netherlands; ^2^Institute for Transfusion Medicine, Hannover Medical SchoolHannover, Germany

**Keywords:** tissue injury, inflammation, cytoprotective molecules, protective immunity, cytoprotective enzymes

In response to tissue injury acute inflammatory reactions occur that aim to restore homeostasis (Medzhitov, 2008). However, hampered resolution of inflammation can result in chronic inflammation and/or pathologic wound repair (Nathan and Ding, 2010). These conditions can result from excessive oxidative and inflammatory and/or overwhelmed adaptive response systems. They may also be triggered by a variety of other conditions including diabetes, infections, or aging. Targeted up-regulation of cytoprotective systems may be a therapeutic approach to ameliorate exacerbation of injury and prevent pathologic wound repair, fibrosis and/or cancer (Nathan, 2002). Such protective systems include various anti-oxidant, anti-inflammatory, anti-apoptotic molecules and also transporters or channels. In this Frontiers research topic various concepts how specific mechanisms can determine tissue damage or protection and their therapeutic potential in a number of pathological conditions and diseases are discussed.

Tissue damage control is important at different levels to maintain homeostasis of cells, tissues and whole organisms. For example, when immunological reactions are primarily directed against the cause of tissue damage (e.g., pathogenic microbes), excessive collateral damage may occur. In such cases, avoiding additional tissue damage would be more important than elimination of the disease-triggering stimulus (Soares, 2014; Soares et al., 2014). Severe tissue injury can lead to chronic inflammation, fibrosis, disturbed developmental changes and cancer (Reuter et al., 2010), which underscores the need to prevent tissue damage and/or to promote regeneration.

The coordinate immunological functions to maintain tissue homeostasis is orchestrated by a selected group of immune cells (Shalapour and Karin, 2015) such as dendritic cells (Mirzaee et al.) and regulatory T-cells (Lei et al.). Targeted modulation of these regulators may thus give control on the decisive machinery that determines immunity, inflammation, tissue remodeling, and cancer (Sutmuller et al., 2007). Infusion of regulatory T-cells facilitates tissue regeneration by preventing undesired immunological activity and by controlling resident non-immune tissue cells and forms an alternative strategy to dampen tissue injury (Lei et al.).

Mizumura and colleagues describe how autophagy may promote tissue damage or repair and novel developments in its regulation (Mizumura et al.). In particular, the role of selective autophagy in a variety of human diseases and the therapeutic potential of this system is discussed.

Surgery and other types of traumatic injury not only cause inflammatory injury, fibrosis, and scar formation (Brouwer et al., 2015), but are associated with the release of free heme (Wagener et al., 2003a). Heme is the prosthetic group of a number of physiologically important hemoproteins (e.g., hemoglobin, cytochromes or cyclooxygenase). However, when heme is not embedded in apoproteins which occurs in pathophysiological situations such as hemolysis or tissue injury, it can mediate or fuel oxidative, inflammatory, and fibrotic insults and may act as a danger signal (Nath et al., 1992; Wagener et al., 2003a,b; Lundvig et al.; Wegiel et al., 2015). High levels of free heme may contribute to or exacerbate tissue injury for example by promoting adhesion molecule expression and leukocyte recruitment (Wagener et al., 2001, 2003a; Belcher et al., 2010; Larsen et al., 2010; Gozzelino and Soares, 2011; Zenclussen et al., 2011). Therefore, protective mechanisms against free heme such as neutralization by either intra- or extra-cellular heme-binding proteins (e.g., hemopexin) or enzymatic heme-degradation by heme oxygenases (HOs) may have important protective functions (Immenschuh et al., 1995; Wagener et al., 2003b, 2013; Kartikasari et al., 2009).

Previously, it has been shown that exposure to small injurious stress stimuli is protective against a follow-up stronger stress. This so-called preconditioning may thus have beneficial effects to protect tissues against injury by promoting cytoprotective signals (Murry et al., 1986). It has been shown that this tissue protection is associated with the induction of cytoprotective genes, such as HO-1, A20, hemopexin, and biliverdin reductase (BVR) (Keyse and Tyrrell, 1989; Hancock et al., 1998).

A major protection system is made up by the HO-1/BVR module. HO is the rate-limiting enzyme of heme degradation, which produces carbon monoxide, iron, and biliverdin, which is converted into bilirubin by BVR. Induction of these cytoprotective proteins has been shown to prolong graft survival after solid organ transplantation by skewing toward a more tolerant immune system (Wagener et al., 2003b). Moreover, HO-activity attenuates the expression of pro-inflammatory cytokines and vascular adhesion molecules, promoting resolution of inflammation (Wagener et al., 2001; Di Francesco et al., 2009). Similar to transplanted organs, tumors are immunologically different to healthy, non-transformed tissues, suggesting that they may also benefit from these cytoprotective enzymes. Likewise, fetuses in the uterus are expressing maternal and paternal proteins, and are thus comparable with an allotransplant. In this topic, it is described how probing antioxidant genes such as HO and BVR could form novel targets for preventing pathological pregnancies (Ozen et al.) and cancer (Gibbs et al.), respectively. Induction of HO activity or exposure to its effector molecules protect against pathological pregnancies, whereas reduced HO-activity disturbs normal pregnancy (Ozen et al.).

BVR interacts with various protein kinases and is involved in a complex system of regulatory pathways. The effects of BVR not only have an important impact on the pathogenesis of cancer, but targeting of this enzyme may ultimately afford the development of novel therapies in cancer (Gibbs et al.). Although, cytoprotective systems such as HO-1/BVR are generally considered to be beneficial, they may be harmful under particular circumstances. For example, it has been demonstrated that HO-1 is involved in the pathogenesis of cancer, because it can protect tumor cells against immune surveillance (Was et al., 2010). Moreover, HO-1 has recently been suggested to be involved in transforming obesity to diabetes (Jais et al., 2014). Therefore, the effects of such cytoprotective systems appear to be critically dependent on cell-type and tissue-context-specific mechanisms.

Another review in this Frontiers topic by Horbach et al. addresses the role of the nuclear factors upstream stimulatory factor (USFs)-1 and -2 in the context of carcinogenesis and tissue injury. USFs have primarily been considered to be involved in the regulation of metabolism, but have also been shown to be intimately associated with tissue protection and the pathogenesis of cancer. Here, the complexity of USF-1 and -2 regulation by various kinases in carcinogenesis is discussed.

Finally, a feasible approach to afford targeted protection in various pathological conditions is to trigger defined protective pathways with safe plant components. For example, induction of anti-inflammatory pathways with herbal compounds and antioxidants suppressed expression of proinflammatory cytokines in human peripheral blood mononuclear cells (Spatuzza et al.), adipose cells (Zagotta et al.), and dendritic cells (Mirzaee et al.). Interestingly, many dietary and natural compounds have been demonstrated to activate nuclear factor erythroid 2-related factor (Nrf2), which in turn induces a number of protective enzymes, such as HO-1, and promote therapeutic effects in cardiovascular diseases (Barbagallo et al., 2013). However, when translating novel protective strategies from the bench to the clinic possible differences in experimental outcome between animal models, cell lines, healthy controls and patients need to be considered (Dorresteijn et al., 2015).

Better insights into the observed differences between pre-conditioning and post-conditioning in relation to tissue repair could further deepen our understanding of these regulatory pathways. It appears likely that learning more about the molecular mechanisms protecting against tissue damage will enable the development of better strategies to prevent or ameliorate wound repair and promote healthy aging.

## Author contributions

All authors listed, have made substantial, direct and intellectual contribution to the work, and approved it for publication.

### Conflict of interest statement

The authors declare that the research was conducted in the absence of any commercial or financial relationships that could be construed as a potential conflict of interest. The reviewer MD and handling Editor declared their shared affiliation, and the handling Editor states that the process nevertheless met the standards of a fair and objective review.
